# Layperson-Administered Naloxone Trends Reported in Emergency Medical Service Activations, 2020-2022

**DOI:** 10.1001/jamanetworkopen.2024.39427

**Published:** 2024-10-14

**Authors:** Christopher B. Gage, Jonathan R. Powell, Alexander Ulintz, Rebecca E. Cash, Michael S. Lyons, Henry Wang, Ashish R. Panchal

**Affiliations:** 1 Division of Epidemiology, The Ohio State University College of Public Health, Columbus; 2 National Registry of Emergency Medical Technicians, Columbus, Ohio; 3 Department of Emergency Medicine, The Ohio State University, Columbus; 4 Department of Emergency Medicine, Massachusetts General Hospital, Harvard Medical School, Boston

## Abstract

**Importance:**

Layperson-administered naloxone (LAN) is a powerful but incompletely characterized intervention to prevent opioid-related overdose mortality. LAN trends are relevant to policy and strategic planning in naloxone distribution initiatives.

**Objective:**

To assess the 2-year LAN trend for persons in the United States receiving naloxone during emergency medical services (EMS) activations.

**Design, Setting, and Participants:**

This retrospective cross-sectional study was conducted in the United States from June 2020 to June 2022 among 65 621 195 EMS activations from 911 responses, EMS standbys, or when EMS crews functioned in an ambulance intercept role or during mutual aid to another ambulance response. Activations within health care settings and interfacility or medical transports were excluded. Data are from the National Emergency Medical Services Information System (NEMSIS), the national EMS patient care record database. From June 2020 to June 2022, NEMSIS included more than 96 million EMS activations from nearly 14 000 agencies across 54 states and territories.

**Exposures:**

EMS clinician–reported LAN.

**Main Outcome and Measures:**

The primary outcome was the trend of receiving LAN, measured by EMS clinician documentation.

**Results:**

From June 2020 to June 2022, EMS reported 744 078 patients receiving naloxone, with 24 990 (3.4%) involving LAN. Patients were predominantly male (17 331 [69.4%]) and had a median (IQR) age of 42 (31-56) years, with the majority treated in urban homes or residences (21 692 [86.8%] urban; 13 223 [52.9%] in-home or residence). Of the total naloxone recipients, 243 985 patients (32.8%) had suspected drug overdose documentation as either the primary or secondary impression. Overall, the percentage change in naloxone administration rates decreased 6.1% over the study period (from 1140.1 [95% CI, 1135.1-1145.1] per 100 000 EMS activations to 1070.1 [95% CI, 1064.9-1075.3] per 100 000 EMS activations), while the percentage change of persons receiving LAN increased 43.5% (from 30.0 [95% CI, 29.2-30.8] per 100 000 EMS activations to 43.1 [95% CI, 42.0-44.1] per 100 000 EMS activations).

**Conclusions and Relevance:**

In this cross-sectional study, the LAN rate increased from June 2020 to June 2022 as reported in the national EMS database. These findings help inform policies and practices aimed at mitigating the devastating impacts of the opioid epidemic and saving lives. Novel public health strategies are needed to measure the effects of this intervention nationally, evaluate approaches to expand naloxone distribution, and address naloxone usage barriers.

## Key Points

**Question:**

Did the frequency of layperson-administered naloxone in conjunction with emergency medical services (EMS) activations change between June 2020 and June 2022?

**Findings:**

In this cross-sectional evaluation of a national dataset of EMS activations in the United States from June 2020 to June 2022, although the rate of EMS-documented naloxone administrations decreased by 6.1%, layperson-administered naloxone before EMS arrival increased by 43.5%.

**Meaning:**

In this study, layperson-administered naloxone drastically increased over the study period, demonstrating the impact of take-home naloxone initiatives and the utility of EMS data to evaluate trends in layperson administration.

## Teaser

This cross-sectional study investigates changes in the rate of layperson-administered naloxone using data from a national US database of emergency medical services (EMS) activations.

## Introduction

The opioid overdose epidemic is a significant public health issue, with fatalities increasing across the United States.^1,2,3^ Naloxone plays an essential role as a lifesaving medication by reversing opioid overdose effects, marking it as a fundamental component in the opioid crisis battle. A recent study reported that in nearly 40% of overdose deaths, there were documented bystanders present who could have potentially given naloxone.^4^

Layperson-administered naloxone (LAN) offers an important rescue option for acute opioid overdose, with distribution programs suggesting LAN can reduce opioid overdose deaths.^5^ Several key steps are now in place to increase LAN within communities^6^; however, legal barriers have curtailed their implementation. Between 2012 and 2016, significant policy changes helped eliminate these barriers and enhance the public availability of naloxone.^7^ However, due to the slow implementation pace in many communities and the substantial public health necessity, the surgeon general issued a public health advisory in 2018 to underline the importance of naloxone usage, the urgent need to educate the public on its use, and the need to enhance the availability of take-home naloxone.^1^ Although these national efforts have boosted awareness and access, evaluating the effectiveness of these programs remains challenging. A national estimate of LAN has yet to be quantified.

Gaining insights from real-time data may be crucial for crafting public health strategies to inform intervention design to reduce mortality rates. Specifically, emergency medical service (EMS) data collection may reflect current LAN use because it captures instances of LAN from first responders who arrive on the scene shortly after an overdose. These data may offer a gauge of LAN trends and effectiveness compared with other sources that might not systematically capture or distinguish between layperson and professional administration events, shedding light on the evolving dynamics of opioid overdose across different communities while evaluating the effectiveness of LAN within these settings. Recognizing this gap, we aim to ascertain the trend of LAN using a national EMS dataset. Using this infrastructure, we can estimate LAN trends over time and better understand their association with the overall opioid epidemic.

## Methods

### Study Design, Setting, and Population

This retrospective observational study assessed persons receiving LAN reported by EMS clinicians in the National Emergency Medical Services Information System (NEMSIS) dataset from June 2020 to June 2022. The American Institutes of Research Institutional Review Board deemed the study exempt from review and the requirement for informed consent because data were deidentified and publicly available. This report follows the Strengthening the Reporting of Observational Studies in Epidemiology (STROBE) reporting guideline.^8^

NEMSIS serves as the national repository for standardized EMS patient care records across US states, offering a framework for collecting, storing, and disseminating these data.^9^ Recognized as the universal standard for capturing patient care information from prehospital 911 calls, NEMSIS was established in 2001 through the collaboration of the National Association of State EMS Directors, the National Highway Traffic Safety Administration, and the Trauma/EMS Systems program of the Health Resources and Services Administration’s Maternal Child Health Bureau. The primary aim of NEMSIS is to enhance the quality and efficiency of EMS through standardized data collection, aggregation, and public usage for quality improvement, research, and policy development.^9^ From June 2020 to June 2022, the system documented more than 96 million EMS activations from 13 946 EMS agencies spanning 54 states and territories.^10,11^ NEMSIS comprises 585 data elements, 165 of which are mandatory at the national level, covering clinical care metrics (such as dispatch and scene times) and activities (including procedures and medications) from dispatch to patient disposition.^12^ The dataset represents EMS operations for approximately 95% of EMS agencies nationwide that handle 911 calls for emergency care and transport to acute care facilities. The NEMSIS Technical Assistance Center receives data from 75% of all electronic patient care reports (ePCRs) generated daily in the United States, with more than 99% of ePCRs submitted within 10 days of patient contact.^13^

EMS activations in this study included 911 responses, EMS standbys, and when EMS crews functioned in an ambulance intercept role or during mutual aid to another ambulance response. Activations were excluded when opioid analgesics were administered, no patient was found, the incident location was a health care facility, or the event was a medical or interfacility transport.

To provide a contextual framework for the naloxone administration rates observed during the study, we also gathered data on opioid overdose mortality rates from the US Centers for Disease Control and Prevention’s (CDC) National Vital Statistics System from 2020 to 2022. We used the Wide-Ranging Online Data for Epidemiologic Research (WONDER), an online system that makes the CDC’s information resources available to health professionals and the public.^14^ By comparing the trends observed in the NEMSIS dataset with those in the CDC WONDER dataset, we aimed to contextualize the EMS records. The consistent trends between the naloxone rates in the NEMSIS data and the opioid-related deaths in the CDC WONDER data provided cross-validation, mitigating potential reporting biases. This dual-dataset approach offers a more comprehensive and reliable analysis, reflecting an accurate account of national opioid overdose trends.

### Main Outcome

The objective of this study was to evaluate the overall trend of patients receiving LAN using EMS-reported data. Naloxone administration events were identified in the EMS patient care report when at least 1 naloxone treatment was documented during any phase of the EMS activation. We determined the incidence of naloxone administration and then stratified the data by the role and type of individual administering the medication. Layperson is defined according to the NEMSIS version 3.4.0 guideline. This variable, “eMedications.10—Role/Type of Person Administering Medication,” lists “Patient/Lay Person (9905023)” as a discrete option for EMS clinicians to document someone not affiliated with an agency or with a known medical background. LAN was defined as instances in which the EMS patient care report indicated that naloxone was administered by a layperson before EMS arrival.

### Measures

Demographic characteristics related to the analysis included patient age categorized as 0 to 14, 15 to 24, 25 to 34, 35 to 44, 45 to 54, 55 to 64, 65 to 74, and 75 years or older and biological sex (male or female). Our dataset had approximately 50% of data on patient race and ethnicity missing, so we did not include these in our analyses. Incident locations are recorded with *International Statistical Classification of Diseases and Related Health Problems, Tenth Revision *(*ICD-10*) in NEMSIS and were categorized as previously described into home or residence, health care facility, non–health care business, street or highway, and other (eg, sporting event, outdoors). Median EMS system response time was computed as the minutes between when the ambulance was notified by dispatch and the time they arrived on the scene.

NEMSIS classifies population setting (urbanicity) using the US Department of Agriculture and Office of Management and Budget definitions using Urban Influence Codes (UIC): urban (UIC 1 and 2), counties with large (≥1 million residents) or small (<1 million residents) metropolitan areas; suburban (UIC 3 and 5), micropolitan (with an urban core of at least 10 000 residents) counties adjacent to a large or small metropolitan county; rural (UIC 4, 6, 8, 9), nonurban core counties neighboring a large metropolitan area or a small metropolitan area (with or without a town); and wilderness (UIC 7, 10, 11, 12), noncore counties that are adjacent to micropolitan counties (with or without own town).^15,16^

The National Center for Health Statistics Mortality Data on CDC WONDER provided data regarding opioid-involved deaths, calculated per 100 000 population. We examined the most recent final multiple causes of death data involving opioid deaths from 2020 to 2022.^17,18^ We focused on cases with underlying causes of death linked to opioid poisoning and involving multiple opioid-related causes on an annual basis. To accurately estimate the incidence of opioid-involved fatalities, the analysis used the *ICD-10* codes X40 to X44, X60 to X64, X85, and Y10 to Y14 to identify the underlying causes of death. Additionally, to gain a more comprehensive understanding of these deaths, we applied *ICD-10* codes T40.0, T40.1, T40.2, T40.3, T40.4, and T40.6 to ascertain the multiple causes of death related to opioid use.

### Statistical Analysis

To assess the overall trend of patients suspected of having opioid overdose by EMS, we identified drug ingestion based on primary and secondary impressions recorded by EMS clinicians using *ICD-10* codes referring to opioids (eg, heroin, opioid, opium, narcotic) (eAppendix 3 in Supplement 1). Because laypersons may not have previous medical training, there is a potential that naloxone is administered to a patient who is presenting with overdose-like symptoms but is experiencing another emergency not related to an overdose (eg, diabetic coma or cardiac arrest). Therefore, not all patients who received naloxone also had a suspected overdose, as defined by the EMS clinician’s impression in the EMS report.

We used descriptive statistics, including mean, median, SD, and IQR, to evaluate patient characteristics, the incidence of total naloxone administrations, and drug-related impressions reported by EMS clinicians. These analyses were conducted with Stata MP version 18 (StataCorp).

We used joinpoint regression to identify specific timeframes in which linear trend shifts occurred in our study (Joinpoint version 5.2.0 [National Cancer Institute]). We analyzed monthly frequencies, assessing the crude rate of LAN per 100 000 EMS 911 activations. Log-linear models were used for linear trends to estimate the monthly percentage change (MPC). Joinpoint calculated a summary estimate for nonlinear trends, the average MPC, using a weighted Bayesian information criterion average of slope coefficients from log-linear models. The empirical quantile method was used to calculate 95% confidence intervals and *P* values for MPCs.^19^ Statistical analysis was set at α = .05.

## Results

### Study Population Characteristics

**Figure 1.**
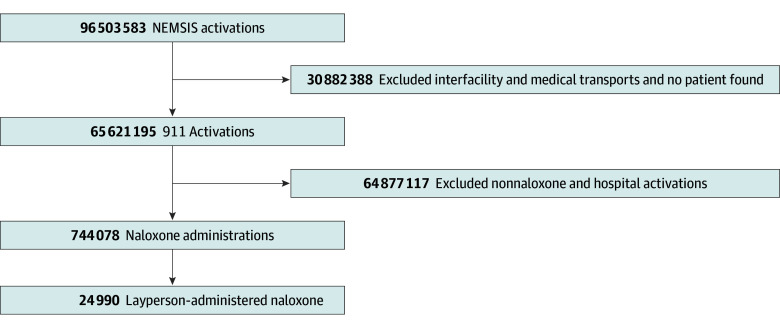
Flow Diagram for Patient Inclusion and Exclusion Criteria From the Complete June 2020 to June 2022 National Emergency Medical Services Information System (NEMSIS) Dataset

Between June 2020 and June 2022, there were more 96 million NEMSIS activations, with 65 621 195 EMS 911 activations in the United States (Figure 1). Among these activations, naloxone was administered to 744 078 patients. Laypersons administered naloxone to 24 990 patients, accounting for 3.4% of all EMS activations with naloxone administration. The patients receiving LAN were primarily male (17 331 [69.4%]) and aged 25 to 34 years (9121 [36.5%]), with a median (IQR) age of 42 (31-56) years (Table 1). Most patients receiving LAN were in urban settings (21 692 [86.8%]) and in a home or residence (13 223 [52.9%]). The median (IQR) EMS system response time for activations with LAN was 6 (4-9) minutes (Table 1). For patients receiving LAN, an overdose impression was documented 14 485 times (58.0%) (Table 1), 27.8% more often than among EMS clinician administrations (eAppendix 1 in Supplement 1). Patients were younger when receiving LAN (median [IQR] age, 35 [29-44] years) than when EMS clinicians administered naloxone (median [IQR] age, 42 [31-57] years). Although the total number of NEMSIS activations increased over the study period, the proportion of EMS activations and patient characteristics stayed the same (eAppendix 2 in Supplement 1).

**Table 1.** Demographic Characteristics of Activations and Patients in Which Layperson-Administered Naloxone Was Reported on an Emergency Medical Services (EMS) Patient Care Report in the National EMS Information System From June 2020 to June 2022

Characteristic	Patients, No. (%)		
	2020 (n = 5225)	2021 (n = 13 340)	2022 (n = 6425)
Age, y			
Median (IQR)	34 (28-42)	35 (29-44)	35 (29-45)
0-14	5 (0.1)	19 (0.1)	17 (0.3)
15-24	651 (12.5)	1589 (12.0)	704 (11.0)
25-34	2058 (39.5)	4785 (36.0)	2278 (35.6)
35-44	1399 (26.9)	3752 (28.2)	1797 (28.1)
45-54	612 (11.8)	1748 (13.1)	916 (14.3)
55-64	391 (7.5)	1092 (8.2)	518 (8.1)
65-74	78 (1.5)	271 (2.0)	145 (2.3)
>74	11 (0.2)	41 (0.3)	24 (0.4)
Missing, No.	20	43	26
Biological sex			
Female	1604 (30.9)	4045 (30.5)	1911 (29.8)
Male	3590 (69.1)	9237 (69.5)	4504 (70.2)
Missing, No.	31	58	10
Urbanicity			
Urban	4561 (90.0)	11 580 (89.5)	5551 (87.9)
Rural	158 (3.1)	452 (3.5)	252 (4.0)
Suburban	304 (6.0)	803 (6.2)	452 (7.2)
Wilderness	42 (0.8)	102 (0.8)	62 (1.0)
Missing, No.	160	403	108
Incident location			
Home or residence	2784 (56.2)	6988 (56.6)	3451 (58.2)
Non–health care business	1148 (23.2)	2851 (23.1)	1444 (24.3)
Street or highway	803 (16.2)	1930 (15.6)	783 (13.2)
Other (eg, sporting events, outdoors)	215 (4.3)	572 (4.6)	255 (4.3)
Missing, No.	275	999	492
Drug OD impression	3106 (59.4)	7798 (58.5)	3581 (55.7)
EMS system response time, median (IQR), min	7 (5-9)	6 (4-9)	6 (5-10)

Abbreviation: OD, overdose.

### Trends in Naloxone Administration

**Figure 2.**
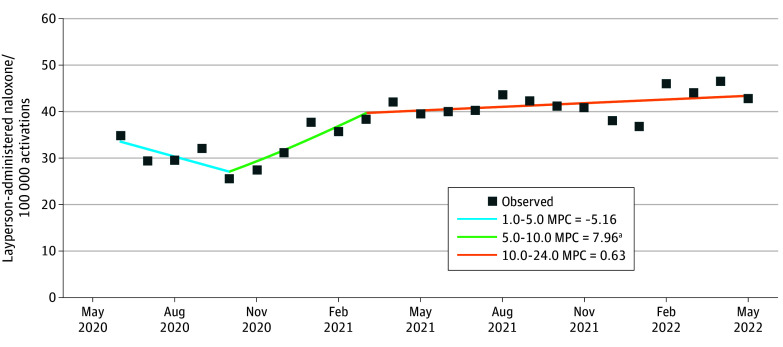
Calculated Crude Rate of Monthly Layperson-Administered Naloxone Against Overall 911 Activations in the National Emergency Medical Services Information System dataset from June 2020 to June 2022 Each square data point signifies the observed crude rates of layperson-administered naloxone. ^a^Indicates that the monthly percentage change (MPC) significantly differs from zero at the α < .05 level.

Although the overall percentage change in naloxone administration rates decreased by 6.1% over the study period (from 1140.1 [95% CI, 1135.1-1145.1] per 100 000 EMS activations to 1070.1 [95% CI, 1064.9-1075.3] per 100 000 EMS activations), LAN saw a 43.5% increase (from 30.0 [95% CI, 29.2-30.8] per 100 000 EMS activations to 43.1 [95% CI, 42.0-44.1] per 100 000 EMS activations) (Table 2). Interestingly, while the rates of overall naloxone administrations and suspected overdose increased between 2020 and 2021, the rate of LAN consistently rose each year. Despite those rising LAN rates, opioid-involved deaths reported by the CDC also increased by 15.2% from 2020 to 2022. Joinpoint regression analysis revealed one significant shift in the LAN trend (Figure 2) occurring between October 2020 and March 2021, with a substantial monthly percentage increase of 7.96% (95% CI, 2.22%-16.92%; *P* = .04). The average MPC of LAN significantly increased throughout the study period by 1.13% (95% CI, 0.45%-1.97%; *P* = .001).

**Table 2.** Incidence Rates and Percentage Change of Emergency Medical Services (EMS) Naloxone Administration Events and Age-Adjusted Opioid Mortality Rate From the National EMS Information System (NEMSIS) and Centers for Disease Control (CDC) National Vital Statistics System, United States, June 2020 to June 2022^a^

Year	NEMSIS EMS reported naloxone administration activations rate (95% CI)^b^			CDC opioid-involved death rate (95% CI)^e^
	Overall naloxone administration (95% CI)	Overall suspected opioid overdose (95% CI)^c^	Layperson-administered naloxone (95% CI)^d^	
2020	1140.1 (1135.1 to 1145.1)	371.6 (368.7 to 374.4)	30.0 (29.2 to 30.8)	28.3 (28.1 to 28.5)
2021	1159.3 (1155.6 to 1162.9)	380.8 (378.7 to 382.9)	40.1 (39.4 to 40.8)	32.2 (32.2 to 32.6)
2022	1070.1 (1064.9 to 1075.3)	352.1 (349.1 to 355.2)	43.1 (42.0 to 44.1)	32.6 (32.4 to 32.8)
% Change^f^	−6.1	−5.2	43.5	15.2

^a^ Naloxone administration event rates are expressed per 100 000 EMS activations; the age-adjusted mortality rate is expressed per 100 000 persons.

^b^ Per 100 000 EMS activations. Data from NEMSIS, June 2020 to June 2022.

^c^ EMS records with primary and secondary impressions were included if any of the *International Statistical Classification of Diseases and Related Health Problems, Tenth Revision *codes documented were for drug ingestion, poisoning, or overdose.

^d^ Patients with EMS-reported layperson administered naloxone (eMedications_03 and eMedications_10) per 100 000 EMS activations. Data from NEMSIS, June 2020 to June 2022.

^e^ Per 100 000 population. Data from CDC’s National Vital Statistics System, Current Final Multiple Cause of Death Data, 2020–2022; CDC WONDER. To obtain estimates of opioid-involved deaths from the Multiple Cause of Death Data, see the WONDER database with *International Statistical Classification of Diseases and Related Health Problems, Tenth Revision * codes X40 to X44, X60 to X64, X85, and Y10 to Y14 for underlying cause of death and *International Statistical Classification of Diseases and Related Health Problems, Tenth Revision *codes T40.0, T40.1, T40.2, T40.3, T40.4, and T40.6 for multiple cause of death.

^f^ Percentage change was calculated as the 2022 rate minus the 2020 rate, divided by the 2020 rate and multiplied by 100.

## Discussion

From June 2020 to June 2022, the United States witnessed a substantial volume of overall EMS activations, within which naloxone played a crucial role in combating opioid overdoses. Although we observed a decrease in the overall naloxone administration trend, LAN experienced particularly significant growth following the onset of the COVID-19 pandemic. This period had a significant increase in LAN usage, underscoring its evolving role in public health strategies aimed at effectively addressing the opioid crisis. These lifesaving interventions, facilitated by legislative changes making naloxone more accessible, such as making naloxone available over the counter or through Good Samaritan laws, increasingly involved laypersons and reflected an important shift toward community involvement in overdose response.^20,21^ The demographics of individuals receiving LAN primarily included young males in urban and residential settings, emphasizing the potential utility of targeted public health strategies.

In the last 10 years, numerous organizations across the United States have enhanced public awareness about opioids and improved the accessibility of naloxone for laypersons. In a 2013 study, 93 organizations distributed or prescribed naloxone to 37 920 laypersons, and of the 68 organizations that collected documentation, they reported 8032 overdose reversals.^22^ As of December 2018, 46 states and the District of Columbia provided legal immunity for friends, family, and other bystanders, or Good Samaritans, seeking medical aid for someone experiencing an opioid overdose.^23^ In 2023, the US Food and Drug Administration approved naloxone hydrochloride nasal spray for over-the-counter, nonprescription use—the first naloxone product approved for use without a prescription.^20^ Early administration is critical in many cases, as evidenced by one study,^24^ which found layperson intervention preceded EMS by 5 minutes or more in 59.5% of cases. In 52.7% of those cases (39 of 74), patients recovered without requiring hospital transportation.^24^ Several studies have found that take-home naloxone programs can be implemented safely, allowing this lifesaving medication to be used by laypersons in locations where the chance of overdose is highest.^25,26,27^

In addressing the critical role of LAN in opioid overdose interventions, it is imperative to delineate the conceptual frameworks underpinning its effectiveness. The distinction between witnessed and unwitnessed overdoses, the presence of bystanders, and the subsequent administration of naloxone, alongside its effectiveness, forms the core of this discussion. Considering the time-sensitive nature of opioid overdose interventions, prompt administration of naloxone by bystanders can be a pivotal determinant of survival.^28^ This delineation is crucial as it emphasizes that the primary goal of LAN is not to address the challenges associated with unwitnessed overdoses but to enhance the outcomes of witnessed overdoses where immediate action by present bystanders can bridge the critical time gap before EMS arrival. The effectiveness of LAN thus lies in its ability to act swiftly within this window, potentially reversing overdose effects and saving lives. By clarifying these conceptual pieces, we underscore the significant role of LAN within the broader context of opioid overdose response strategies, explicitly highlighting its lifesaving potential in scenarios where timely intervention is possible and critical.

Our study uniquely focuses on cases where 911 was called, a strength in that it highlights situations likely involving bystanders who could administer naloxone but also limits our scope to these scenarios. This design highlights the uneven distribution and varying ease of naloxone use among the public. While an optimal harm-reduction approach would see bystander naloxone administration nearing 100%, our findings reveal a lower-than-ideal utilization rate accounting for only 3.4% of all EMS activations with naloxone administration. This gap underscores the imperative to not only improve naloxone education and access but also to investigate other obstacles to its utilization in real-world contexts.^29^

Our findings on LAN amid the opioid crisis underscore the necessity of considering system-level implications, particularly in light of evolving drug markets—from heroin to the more potent fentanyl—and the dynamics of naloxone availability and prescribing practices.^30^ The transition in the drug landscape not only heightens the urgency for naloxone as a critical intervention but also suggests a comprehensive strategy that integrates enhanced accessibility and targeted education on naloxone use. This approach must be adaptive to the changing nature of opioid misuse and equipped to address the barriers that currently limit naloxone’s use among those most at risk. Ultimately, our study not only highlights the underutilized potential of naloxone in combating opioid overdoses but also serves as a call to action for policymakers, health care leaders, and communities to refine and expand harm reduction efforts at a systemic level, ensuring that naloxone can fulfill its lifesaving promise in the face of an evolving opioid epidemic.

### Limitations

This evaluation is subject to several limitations that impact the accuracy of depicting the landscape of LAN in the United States. Underreporting likely excludes less severe overdose cases that do not require EMS intervention, potentially underestimating the prevalence of LAN.^29,31^ Despite this, our study provides a crucial first step in establishing national estimates for LAN, highlighting its importance. The absence of a unified tracking system for naloxone administration outside formal medical settings inhibits our understanding of its usage patterns. However, this underscores the need for future systems to enhance LAN monitoring.

Although NEMSIS reports 99% of ePCRs are submitted within 10 days of patient contact,^13^ delays from EMS activation to data reporting could introduce recall bias, and incomplete data entry within EMS records may affect the reliability of our findings. The inability to confirm opioid intoxication due to the patient’s state can cause potentially inaccurate EMS assessments and further complicate data accuracy. Additionally, no current studies verify the accuracy of naloxone administration coding with *ICD-10* codes in NEMSIS. Nonetheless, our study draws attention to these areas, paving the way for improvements in data collection and assessment methods.

Our dataset limitations prevent analysis of patient outcomes after LAN, and the significant missing race and ethnicity data (approximately 50%) limits the evaluation of these trends across diverse communities. Nevertheless, identifying these limitations highlights the need for more inclusive data collection efforts. Additionally, a potential challenge in LAN measurement is whether documented administration is simply a response to overdose trends rather than improved naloxone access or utilization by laypersons. In contrast, while we note an overall decrease in naloxone administration reported by EMS since 2020, LAN use has continually increased. These limitations suggest a cautious interpretation of our results and highlight areas for further research to address these gaps while celebrating the progress made in understanding LAN.

## Conclusions

Our study highlights the increasing trend of LAN in the face of the United States’ opioid crisis, revealing significant increases in its use, especially during periods following the COVID-19 pandemic onset. These findings emphasize the evolving role of LAN in public health strategies, stressing the importance of community involvement in emergency overdose responses and the need for targeted education and enhanced accessibility to naloxone. As the landscape of opioid misuse continues to shift, our research underscores the urgent need for comprehensive strategies that not only increase naloxone availability but also address current barriers to its use, maximizing its potential to save lives amid an ongoing opioid epidemic.

## Notes

Supplement 1. **eAppendix 1.** Demographic Characteristics of Activations and Patients Where EMS Clinician-Administered Naloxone Was Reported on an Emergency Medical Services (EMS) Patient Care Report **eAppendix 2.** Evaluation of Annual Patient Characteristics for the Complete National Emergency Medical Services System Information (NEMSIS) Dataset for 2018-2022 **eAppendix 3.** International Classification of Diseases (ICD) Tenth Edition Codes and Descriptors Used for the Primary and Secondary Impressions of Opioid Overdose

Supplement 2.
Data Sharing Statement
