# Supplementary material for: Layperson-Administered Naloxone Trends Reported in Emergency Medical Service Activations, 2020-2022
Source: JAMA Netw Open. 2024 Oct 14;7(10):e2439427. doi: 10.1001/jamanetworkopen.2024.39427 (PMC11581548; doi:10.1001/jamanetworkopen.2024.39427)
Supplement: Supplement 1. — eAppendix 1. Demographic Characteristics of Activations and Patients Where EMS Clinician-Administered Naloxone Was Reported on an Emergency Medical Services (EMS) Patient Care Report eAppendix 2. Evaluation of Annual Patient Characteristics for the Complete National Emergency Medical Services System Information (NEMSIS) Dataset for 2018-2022 eAppendix 3. International Classification of Diseases (ICD) Tenth Edition Codes and Descriptors Used for the Primary and Secondary Impressions of Opioid Overdose [file jamanetwopen-e2439427-s001.pdf]

Supplemental Online Content

Gage CB, Powell JR, Ulintz A, et al. Layperson-administered naloxone trends reported in emergency medical service activations, 2020-2022. *JAMA Netw Open.* 2024;7(10):e2439427. doi:10.1001/jamanetworkopen.2024.39427

eAppendix 1. Demographic Characteristics of Activations and Patients Where EMS Clinician-Administered Naloxone Was Reported on an Emergency Medical Services (EMS) Patient Care Report

eAppendix 2. Evaluation of Annual Patient Characteristics for the Complete National Emergency Medical Services System Information (NEMSIS) Dataset for 2018-2022

eAppendix 3. International Classification of Diseases (ICD) Tenth Edition Codes and Descriptors Used for the Primary and Secondary Impressions of Opioid Overdose

This supplemental material has been provided by the authors to give readers additional information about their work.

© 2024 Gage CB et al. *JAMA Network Open.*

eAppendix 1. Demographic Characteristics of Activations and Patients Where EMS Clinician-Administered Naloxone Was Reported on an Emergency Medical Services (EMS) Patient Care Report

	EMS Clinician-Administered Naloxone Population				
	2018<br>N=101,197<br>No (%)	2019<br>N=170,467<br>No (%)	2020<br>N=264,599<br>No (%)	2021<br>N=297,850<br>No (%)	2022<br>N=306,309<br>No (%)
Age in years, mean (SD)	47 (19)	46 (18)	45 (18)	46 (18)	47 (18)
Age categories					
0-14	527 (1%)	987 (0%)	1,198 (0%)	1,638 (0%)	1,908 (1%)
15-24	9,525 (10%)	15,224 (9%)	25,068 (10%)	25,845 (9%)	23,176 (8%)
25-34	23,630 (24%)	39,048 (23%)	64,426 (25%)	68,097 (23%)	65,141 (21%)
35-44	18,602 (19%)	32,656 (19%)	55,278 (21%)	64,517 (22%)	67,363 (22%)
45-54	16,178 (16%)	28,014 (17%)	42,338 (16%)	48,094 (16%)	50,371 (17%)
55-64	15,754 (16%)	27,819 (16%)	40,654 (15%)	46,936 (16%)	50,465 (17%)
65-74	8,634 (9%)	14,413 (9%)	19,718 (8%)	24,790 (8%)	27,647 (9%)
≥75	6,833 (7%)	10,717 (6%)	13,655 (5%)	15,436 (5%)	17,188 (6%)
Missing	1,514	1,589	2,264	2,497	3,050
Biological Sex					
Male	61,029 (61%)	106,194 (63%)	171,776 (66%)	194,671 (66%)	201,179 (66%)
Female	38,317 (39%)	61,547 (37%)	88,426 (34%)	98,666 (34%)	101,427 (34%)
Missing	1,851	2,726	4,397	4,513	3,703
Urbanicity					
Urban	84,000 (85%)	144,797 (87%)	225,607 (87%)	255,358 (88%)	264,572 (88%)
Rural	6,467 (7%)	9,596 (6%)	13,856 (5%)	15,531 (5%)	15,797 (5%)
Suburban	6,270 (6%)	9,716 (6%)	15,870 (6%)	16,495 (6%)	16,598 (6%)
Wilderness	1,547 (2%)	2,237 (1%)	3,191 (1%)	3,514 (1%)	3,424 (1%)
Missing	2,913	4,121	6,075	6,952	5,918
Incident location					
Home/Residence	53,897 (63%)	85,854 (59%)	125,524 (58%)	133,231 (56%)	137,277 (55%)

© 2024 Gage CB et al. *JAMA Network Open.*

Non-Healthcare Business	13,844 (16%)	23,284 (16%)	35,731 (17%)	42,313 (18%)	46,242 (19%)
Street or Highway	12,739 (15%)	21,786 (15%)	35,987 (17%)	43,279 (18%)	46,781 (19%)
Other (e.g., sporting events, outdoors)	5,406 (6%)	15,460 (11%)	17,810 (8%)	19,965 (8%)	18,474 (7%)
Missing	15,311	24,083	49,547	59,062	57,535
Drug O.D. impression	54,836 (54%)	96,202 (56%)	156,332 (59%)	172,348 (58%)	178,625 (58%)
Median Response Time	6 (4,9)	6 (4,9)	6 (4,9)	6 (4,8)	6 (4,9)

Abbreviations: IQR – interquartile range, O.D. – overdose, S.D. – standard deviation.

© 2024 Gage CB et al. *JAMA Network Open.*

eAppendix 2. Evaluation of Annual Patient Characteristics for the Complete National Emergency Medical Services System Information (NEMSIS) Dataset for 2018-2022

Abbreviations: CI – confidence interval, IQR – interquartile range.

	2018 (n=22,532,890)<br><br>% (95% CI)	2019 (n = 34,203,087)<br><br>% (95% CI)	2020 (n= 43,440,337)<br><br>% (95% CI)	2021 (n= 48,982,990)<br><br>% (95% CI)	2022 (n= 53,179,492)<br><br>% (95% CI)
EMS Activations	69.3 (69.3-69.4)	68.1 (68.0-68.1)	67.7 (67.7-67.8)	67.9 (67.9-67.9)	69.2 (69.2-69.2)
Age, median (IQR)	56 (34-73)	56 (34-73)	57 (35-73)	57 (35-73)	58 (35-74)
Sex					
Female	43.2 (43.2-43.3)	42.9 (42.9-42.9)	42.5 (42.5-42.5)	42.9 (42.9-42.9)	43.0 (43.0-43.0)
Male	40.4 (40.4-40.5)	40.5 (40.5-40.5)	42.6 (42.6-42.6)	42.2 (42.2-42.2)	41.6 (41.5-41.6)
Missing	5.1 (5.1-5.1)	5.3 (5.3-5.3)	4.8 (4.8-4.8)	4.7 (4.7-4.7)	4.5 (4.5-4.5)
Incident Location					
Home/Residence	41.2 (41.2-41.3)	43.1 (43.0-43.1)	43.0 (43.0-43.0)	41.8 (41.8-41.8)	41.6 (41.6-41.6)
Non-Healthcare Business	12.9 (12.9-12.9)	12.5 (12.5-12.5)	10.2 (10.2-10.2)	10.9 (10.8-10.9)	11.7 (11.6-11.7)
Street or Highway	13.1 (13.0-13.1)	13.0 (13.0-13.0)	12.3 (12.3-12.3)	12.0 (12.0-12.0)	11.5 (11.5-11.5)
Other	4.1 (4.1-4.1)	4.5 (4.5-4.5)	3.4 (3.4-3.4)	3.6 (3.6-3.7)	3.4 (3.4-3.4)
Missing	17.5 (17.5-17.5)	15.6 (15.6-15.6)	21.0 (21.0-21.1)	21.4 (21.4-21.4)	20.9 (20.9-20.9)
Urbanicity					
Rural	5.7 (5.6-5.7)	5.7 (5.7-5.7)	5.7 (5.7-5.7)	5.7 (5.7-5.7)	5.4 (5.4-5.4)
Suburban	4.7 (4.6-4.7)	4.8 (4.7-4.9)	5.3 (5.3-5.3)	5.1 (5.1-5.1)	4.9 (4.9-4.9)
Urban	73.8 (73.8-73.8)	73.6 (73.6-73.6)	74.7 (74.7-74.8)	74.9 (74.9-74.9)	75.2 (75.2-75.2)
Wilderness	1.4 (1.4-1.4)	1.5 (1.5-1.5)	1.4 (1.4-1.4)	1.4 (1.4-1.4)	1.3 (1.3-1.3)
Missing	3.2 (3.2-3.3)	3.0 (3.0-3.0)	2.8 (2.8-2.8)	2.8 (2.7-2.8)	2.3 (2.3-2.3)

© 2024 Gage CB et al. *JAMA Network Open.*

eAppendix 3. International Classification of Diseases (*ICD*) Tenth Edition Codes and Descriptors Used for the Primary and Secondary Impressions of Opioid Overdose
Abbreviation: NOS - not otherwise specified.

ICD-10 Codes	Descriptor
T40.2X5	Adverse effect of other opioids
F11.1	Opioid abuse
F11.12	Opioid abuse with intoxication
F11.121	Opioid abuse with intoxication delirium
F11.122	Opioid abuse with intoxication with perceptual disturbance
F11.120	Opioid abuse with intoxication, uncomplicated
F11.129	Opioid abuse with intoxication, unspecified
F11.15	Opioid abuse with opioid-induced psychotic disorder
F11.18	Opioid abuse with other opioid-induced disorder
F11.19	Opioid abuse with unspecified opioid-induced disorder
F11.10	Opioid abuse, uncomplicated
F11.2	Opioid dependence
F11.222	Opioid dependence with intoxication with perceptual disturbance
F11.229	Opioid dependence with intoxication, unspecified
F11.24	Opioid dependence with opioid-induced mood disorder
F11.29	Opioid dependence with unspecified opioid-induced disorder
F11.23	Opioid dependence with withdrawal
F11.21	Opioid dependence, in remission
F11.20	Opioid dependence, uncomplicated
F11	Opioid related disorders
F11.9	Opioid use, unspecified
F11.92	Opioid use, unspecified with intoxication
F11.921	Opioid use, unspecified with intoxication delirium
F11.922	Opioid use, unspecified with intoxication with perceptual disturbance
F11.920	Opioid use, unspecified with intoxication, uncomplicated

© 2024 Gage CB et al. *JAMA Network Open.*

F11.929	Opioid use, unspecified with intoxication, unspecified
F11.95	Opioid use, unspecified with opioid-induced psychotic disorder
F11.99	Opioid use, unspecified with unspecified opioid-induced disorder
F11.93	Opioid use, unspecified with withdrawal
F11.90	Opioid use, unspecified, uncomplicated
T40.2X1	Poisoning by other opioids, accidental (unintentional)
T40.2X1A	Poisoning by other opioids, accidental (unintentional), initial encounter
T40.2X1S	Poisoning by other opioids, accidental (unintentional), sequela
T40.2X2A	Poisoning by other opioids, intentional self-harm, initial encounter
T40.2X2S	Poisoning by other opioids, intentional self-harm, sequela
T40.2X4	Poisoning by other opioids, undetermined
T40.2X4A	Poisoning by other opioids, undetermined, initial encounter
T40.2	Poisoning by, adverse effect of and underdosing of other opioids
T40.601	Poisoning by narcotics NOS
T40.0X1	Poisoning by opium NOS
T40.0X1A	Poisoning by opium, accidental (unintentional), initial encounter
T40.0X3A	Poisoning by opium, assault, initial encounter
T40.0X2A	Poisoning by opium, intentional self-harm, initial encounter
T40.0X4	Poisoning by opium, undetermined
T40.0X4A	Poisoning by opium, undetermined, initial encounter
T40.691	Poisoning by other narcotics NOS
T40.692	Poisoning by other narcotics, intentional self-harm
T40.692A	Poisoning by other narcotics, intentional self-harm, initial encounter
T40.694	Poisoning by other narcotics, undetermined
T40.694A	Poisoning by other narcotics, undetermined, initial encounter
T40.4X1	Poisoning by other synthetic narcotics NOS
T40.4X1A	Poisoning by other synthetic narcotics, accidental (unintentional), initial encounter
T40.4X2A	Poisoning by other synthetic narcotics, intentional self-harm, initial encounter
T40.4X4	Poisoning by other synthetic narcotics, undetermined
T40.601A	Poisoning by unspecified narcotics, accidental (unintentional), initial encounter

© 2024 Gage CB et al. *JAMA Network Open.*

T40.601D	Poisoning by unspecified narcotics, accidental (unintentional), subsequent encounter
T40.603A	Poisoning by unspecified narcotics, assault, initial encounter
T40.602	Poisoning by unspecified narcotics, intentional self-harm
T40.602A	Poisoning by unspecified narcotics, intentional self-harm, initial encounter
T40.604	Poisoning by unspecified narcotics, undetermined
T40.604A	Poisoning by unspecified narcotics, undetermined, initial encounter
T40.604S	Poisoning by unspecified narcotics, undetermined, sequela
T40.695	Adverse effect of other narcotics
T40.4X5	Adverse effect of other synthetic narcotics
T40.605	Adverse effect of unspecified narcotics
T40.0X5A	Adverse effect of opium, initial encounter
T40.1	Poisoning by and adverse effect of heroin
T40.1X	Poisoning by and adverse effect of heroin
T40.1X1	Poisoning by heroin NOS
T40.1X1A	Poisoning by heroin, accidental (unintentional), initial encounter
T40.1X1S	Poisoning by heroin, accidental (unintentional), sequela
T40.1X3A	Poisoning by heroin, assault, initial encounter
T40.1X2	Poisoning by heroin, intentional self-harm
T40.1X2A	Poisoning by heroin, intentional self-harm, initial encounter
T40.1X2S	Poisoning by heroin, intentional self-harm, sequela
T40.1X4	Poisoning by heroin, undetermined
T40.1X4A	Poisoning by heroin, undetermined, initial encounter
T40.1X4S	Poisoning by heroin, undetermined, sequela

© 2024 Gage CB et al. *JAMA Network Open.*
