# Supplementary material for: Layperson-Administered Naloxone Trends Reported in Emergency Medical Service Activations, 2020-2022
Source: JAMA Netw Open. 2024 Oct 14;7(10):e2439427. doi: 10.1001/jamanetworkopen.2024.39427 (PMC11581548; doi:10.1001/jamanetworkopen.2024.39427)
Supplement: Supplement 2. — Data Sharing Statement [file jamanetwopen-e2439427-s002.pdf]

Data Sharing Statement

Gage. Layperson-Administered Naloxone Trends Reported in Emergency Medical Service Activations, 2020-2022. *JAMA Netw Open*. Published October 14, 2024.
doi:10.1001/jamanetworkopen.2024.39427

Data
Data available: Yes
Data types: Deidentified participant data
How to access data: All data are publically available through the NEMSIS TAC and CDC Wonder database. https://nemsis.org/using-ems-data/request-research-data/
When available: With publication

Supporting Documents
Document types: None

Additional Information
Who can access the data: Publically available.
Types of analyses: At the discretion of the NEMSIS TAC.
Mechanisms of data availability: With investigator support if needed.
Any additional restrictions: All data is deidentified.
